# Evaluation of molecular signatures in the urinary bladder and upper tract urothelial carcinomas: a prospective controlled clinical study

**DOI:** 10.1186/s43046-022-00148-x

**Published:** 2022-11-14

**Authors:** Eman E. Dawood, Amira Awadalla, Abdelwahab Hashem, Ahmed A. Shokeir, A. F. Abdel-Aziz

**Affiliations:** 1 Biochemistry Division, Department of Chemistry, Faculty of Science, Mansoura University, Mansoura, 35516 Egypt; 2 Center of Excellence for Genome and Cancer Research, Urology and Nephrology Center, Mansoura University, Mansoura, 35516 Egypt; 3 Urology Department, Urology and Nephrology Center, Mansoura University, Mansoura, 35516 Egypt

**Keywords:** Bladder cancer, Upper tract, Carcinoma, Molecular pathway, Oncogene

## Abstract

**Background:**

Urothelial carcinomas (UC) can be either in the upper or in the lower urinary tract or both. Urothelial bladder cancer (UBC) is more common than upper tract urothelial carcinoma (UTUC). This research was designed to study the difference between UBC and UTUC using the molecular pathways including (MAPK/ERK) pathway, cell cycle regulating genes, and oncogenic genes.

**Methods:**

To study the discrepancy between UBC and UTUC, a prospective trial was carried out for 31 radical cystectomy and 19 nephrouretrectomy *fresh-frozen* specimens of UBC and UTUC patients, respectively. The expression level of mRNA of eight genes namely EGFR, ELK1, c-fos, survivin, TP53, RB1, FGFR3, and hTERT was assessed in normal adjacent tissues, UTUC, and UBC by RT-PCR.

**Results:**

Comparison between UTUC and UBC regarding the expression level of mRNA of the *EGFR*, *ELK1*, *c-fos*, *survivin*, *TP53*, and *FGFR3* had significant difference (*p*-value < 0.001), while the expression level of *RB1* and *hTERT* level had no significance. Sensitivity/specificity of *EGFR*, *Elk1*, *c-fos*, *survivin*, *TP53*, and *FGFR3* was 0.78/0.90, 0.84/0.90, 0.84/0.80, 0.84/0.96, 0.94/0.93, and 0.89/0.93, respectively, to differentiate between UTUC and UBC.

**Conclusions:**

Despite the fact that UTUC and UBC share the same origin, there is a clear evidence that there is a molecular difference between them. This molecular difference could be the reason that UTUC is more aggressive than UBC.

## Background

Urothelium is the specific epithelial lining of the upper urinary tract that includes the renal pelvis and ureters and lower urinary tract that includes bladder and urethra [1]. Urothelial carcinomas (UC) can be in the upper and/or the lower urinary tract. Urothelial cancers are the most abundant histologic subtype of bladder cancer (BC) with nearly 90% of bladder tumors being urothelial. Upper tract urothelial carcinoma (UTUC) is uncommon, accounting for only 5–10% of all UC [2].

Both UC of the bladder and upper tract are considered as a disparate twin. Both originates from the same tissue type; however, both have discrepancies in diagnosis, treatment, and prognosis [3]. Both arise from distinct embryonic tissues and have overlapping genomic features [4].

Previous studies had reported that UTUC was more aggressive than UBC [5]. The tumor aggressiveness is controlled by MAPK\ERK pathway, cell cycle regulators, and oncogenes as they are the major pathways responsible for the tumor invasiveness and metastasis [6–8].

MAPK/ERK is a critical pathway for human cancer. It plays an critical role in cell proliferation, differentiation, apoptosis, angiogenesis, and tumor metastasis [6]. Mutations and overexpression of these pathway genes are responsible for the regulation of cell fate, genome integrity, and survival leading to protein amplification, thus overactivating the pathway. These mutations can occur in membrane receptor genes, such as epithelial growth factor receptor (*EGFR*), and in transcription factors (*ELK-1* & *c-fos*) which had been studied in both tumors separately in previous studies [9].

Cell cycle pathways involved in carcinogenesis and have a role in tumor prognosis and management. Mutations in cell cycle regulatory genes are the most prevalent in cancer, and they can lead to uncontrolled tumor development and progression. In human urothelial carcinomas, mutations and deregulation of genes involved in the regulation of normal cell cycle progression are common. *TP53*, *retinoblastoma* (*RB1*), and *survivin* gene mutations are common in UBC and UTUC [4, 7, 10].

Proto-oncogenes have vital functions which are essential for normal homeostasis in cells. Proto-oncogenes are responsible of growth, proliferation, and survival. Tumor cells can then make use of an oncogene’s beneficial properties [8]. Mutated *fibroblast growth factor receptor 3 (FGFR3)* and *hTERT* are implicated as the most common oncogenes in the UC [11].

Several studies had compared between UBC and UTUC in epidemiology, etiology, staging, risk factors, prognosis, and management [1, 2]. Regarding molecular level, UBC is more common and better studied in molecular alterations characterization than UTUC, even in the Cancer Genome Atlas (TCGA) project, likely due to the rarity of the UTUC [12]. There are no enough molecular studies to compare between UBC and UTUC [13].

Therefore, this study was designed to investigate the molecular pathways in the two types of UC, through shedding the light on *EGFR*, *ELK1*, and *c-fos* from the (MAPK/ERK) pathway; *survivin*, *TP53*, and *RB1* from the cell cycle regulating genes; and *FGFR3* and *hTERT* from the oncogenic genes.

## Patients and methods

### Patients

This prospective study was performed on pathological specimens of 31 subjects with UBC who underwent cystectomy and 19 with UTUC who underwent radical nephroureterectomy with bladder cuff excision. In each group, fresh-frozen samples were taken from the malignant tissue and non-tumor tissue of the same specimen qRT-PCR. All tumors were graded using the 2004 WHO classification and staged according to the 1997 TNM classification. The institutional review board (IRB) approved this study, and informed consent was taken from all patients.

## Methods

### Real-time quantitative reverse transcription PCR (qRT-PCR)

Total RNA was extracted from frozen tissue specimens using TRIzol (Invitrogen, USA) according to the manufacturer’s protocol. Complementary DNA (cDNA) was synthesized from RNA using high-capacity cDNA reverse transcription kit (Applied Biosystems, USA), following manufacturer’s instructions. qRT-PCR was achieved using SYBER Green PCR Master Mix (Applied Biosystems, USA). The mRNA expression level of *EGFR*, *ELK1*, *c-fos*, *survivin*, *TP53*, *RB1*, *FGFR3*, *hTERT*, and *GAPDH* as the internal control was quantified using step 1 plus real-time PCR (Applied Biosystems). All primers are listed in Table 1. The qRT-PCR program was as follows: initial denaturation for 10 min at 95 °C, then 40 cycles of denaturation for 15s at 95 °C, annealing at 60 °C for 1 min, and finally extension at 72 °C for 1 min. Expression level analysis was done based on the equation (2^−ΔΔCT^) [14].

**Table 1:** List of primer sequence for genes in study

**Gene**	**Sequence**	**Accession no.**
***EGFR***	**F:** 5′GCTGCCAAAAGTGTGATCCAAG3′ **R:** 5′CATGGAGGTCCGTCCTGTTTTC3′	NM_201282.2
***ELK-1***	**F:** 5′ACTCCTCCGCATCCCTCTTT3′ **R:** 5′TCCCGTGAAGTCCAGGAGAT3′	NM_001257168.1
***c-fos***	**F:** 5′GGGGCAAGGTGGAACAGTTAT3′ **R:** 5′CCGCTTGGAGTGTATCAGTCA3′	NM_005252.4
***Survivin***	**F:** 5′TGGCAGCCCTTTCTCAAGGACC3′ **R:** 5′TCGATGGCACGGCGCACTTTCTCC3′	NM_001012270.2
***TP53***	**F:** 5′AATTTGCGTGTGGAGTATTT3′ **R:** 5′CTGGAGTCTTCCAGTGTGAT3′	NM_001126118.2
***RB1***	**F:**5′TGTAATGGCCACATATAGCAGAAGT3′ **R:** 5′TAAGAGGACAAGCAGATTCAAGGTG3′	NM_000321.3
***FGFR3***	**F:** 5′GATGGACAAGAAGCTGCTGG3′ **R:** 5′TGCCAAACTTGTTCTCCACG3′	NM_001163213.2
***hTERT***	**F:** 5′GCACTGGCTGATGAGTGTGT3′ **R:** 5′CTCGGCCCTCTTTTCTCTG3′	NM_001193376.3
***GAPDH***	**F:** 5′TGCTGGCGCTGAGTACGTCG3′ **R:** 5′TGACCTTGGCCAGGGGTGCT3′	NM_001357943.2

### Statistical analysis

Statistical analysis was performed with the use of the IBM SPSS Statistics ver. 22.0 for Windows (IBM Corp., Armonk, NY, USA). In the normally distributed variables, student *t*-test was used for comparison between groups. Chi-square and Fisher exact tests were used for comparing categorical data of both groups. Receiver operating characteristic analysis (ROC), an area under the curve (AUC), sensitivity, and specificity were calculated. *p*-value ≤ 0.05 was considered statistically significant.

## Results

Thirty-one subjects with UBC and 19 subjects with UTUC were included in this study. Table 2 shows the patient demographic characteristics of the two groups. Groups were matched in age, sex, BMI, and renal function. Moreover, comorbidities in terms of liver disease, diabetes mellitus, and chronic kidney disease were not significantly different among both groups.

**Table 2:** Demographic and oncologic features of UTUC and UBC

	**UTUC**	**UBC**	*p***-value**
**Age, years (mean ± SD)**	63.89 ± 10.21	61.9 ± 10.64	0.517
**BMI (mean ± SD)**	29.17 ± 6.5	30.97 ± 7.63	0.396
**Gender, no. (%)**			
**Male**	16 (84.2%)	25 (80.6%)	0.750
**Female**	3 (15.8%)	6 (19.4%)	
**Comorbidities, no. (%)**			
**Liver disease**			
**Yes**	0	4 (12.9%)	0.103
**No**	19 (100%)	27 (87.1%)	
**Diabetes mellitus**			
**Yes**	2 (10.5%)	10 (32.3%)	0.081
**No**	17 (89.5%)	21 (67.7%)	
**CKD**			
**Yes**	1 (5.3%)	3 (9.7%)	0.577
**No**	18 (94.7%)	28 (90.3%)	
**Serum creatinine, mg/dL (mean ± SD)**	1.31 ± 0.5	1.3 ± 1.2	0.994
**T stage, no. (%)**			
**T1**	17 (89.5%)	7 (22.6%)	< 0.001*
**T2**	1 (5.3%)	8 (25.8)	
**T3**	1 (5.3%)	13 (41.9)	
**T4**	0	3 (9.7%)	
**N stage, no. (%)**			
**N0/×**	16 (84.2%)	18 (58.1%)	0.107
**N1 (single)**	3 (15.8%)	5 (16.1%)	
**N2 (multiple)**	0	6 (19.4%)	
**N3**	0	2 (6.5%)	
**Organ confined, no. (%) (T stage ≤ 2, N0/×)**	16 (84.2%)	12 (38.7%)	0.002*
**Non-organ confined, no. (%) (T stage > 2, N0/×/+)**	3 (15.8%)	19 (61.3%)	
**Cell type final pathology, no. (%)**			
**Pure TCC**	19 (100%)	21 (67.7%)	0.105
**TCC with squamous differentiation**	0	7 (22.6%)	
**TCC with micropapillary component**	0	1 (3.2%)	
**TCC with sarcomatoid differentiation**	0	1 (3.2%)	
**TCC with squamous and sarcomatoid differentiation**	0	1 (3.2%)	
**Grade,***n***(%)**			
**Low grade**	0	1 (3.2%)	0.429
**High grade**	19 (100%)	30 (96.8%)	
**Lymphovascular invasion final pathology, no. (%)**			
**Yes**	14 (73.7%)	13 (41.9%)	0.029
**No**	5 (26.3%)	18 (58.1%)	

*p ≤ 0.05

Oncologic comparison between both groups is shown in Table 2. UTUC had significantly lower tumor stage with 89.5% having T1. Moreover, 84.2% of UTUC had organ confined ≤ T2, N0/× versus 38.7% in UBC, a difference of statistical significance. On the hand, lymphovasular invasion was significantly higher in UTUC 73.7% versus 32% in UBC. Meanwhile, both groups were comparable regarding tumor grade, lymph node status, and pathological cell type.

### Gene expression

#### MAPK/ERK pathway

Comparison between the expression level of mRNA of MAPK/ERK pathway including *EGFR*, *ELK-1*, and *c-fos* showed significantly higher levels of expression in both UTUC and UBC compared to normal tissue (*P* < 0.001). Comparison between UTUC and UBC showed significantly higher levels of expression of the three genes in UTUC (*P* < 0.001) (Table 3).

**Table 3:** Comparison of genes expression between UTUC and UBC

	**UTUC**	**Noncancerous upper tract**	**UBC**	**Noncancerous bladder**	*p***-value**
**MAPK/ERK pathway**					
**EGFR**	5.28 ± 2.69	1.05 ± 0.9	2.85 ± 0.9	1.05 ± 0.14	< 0.001^a, b, c^
**ELK-1**	5.00 ± 0.97	0.97 ± 0.08	3.15 ± 0.72	1.07 ± 0.18	< 0.001^a, b, c^
**c-fos**	5.11 ± 1.85	1 ± 0.07	3.12 ± 0.77	1.06 ± 0.16	< 0.001^a, b, c^
**Cell cycle regulators**					
**Survivin**	12.75 ± 6.28	0.98 ± 0.1	5.73 ± 1.32	1.02 ± 0.15	< 0.001^a, b, c^
**TP53**	5.75 ± 1.21	0.96 ± 0.07	3.21 ± 0.8	1.03 ± 0.97	< 0.001^a, b, c^
**RB1**	0.37 ± 0.37	1.03 ± 0.08	0.50 ± 0.25	1.01 ± 0.09	< 0.001^a, b^ 0.133^c^
**Oncogenic genes**					
**FGFR3**	5.12 ± 2.82	1 ± 0.07	2.88 ± 0.60	1.06 ± 0.16	< 0.001^a, b, c^
**hTERT**	3.56 ± 1.3	1.02 ± 0.09	3.28 ± 0.97	1.02 ± 0.1	< 0.001^a, b^ 0.395c

^a^*p*-value between UTUC and noncancerous upper tract

^b^*p*-value between UBC and noncancerous bladder

^c^*p*-value between UTUC and UBC

**Fig. 1:**
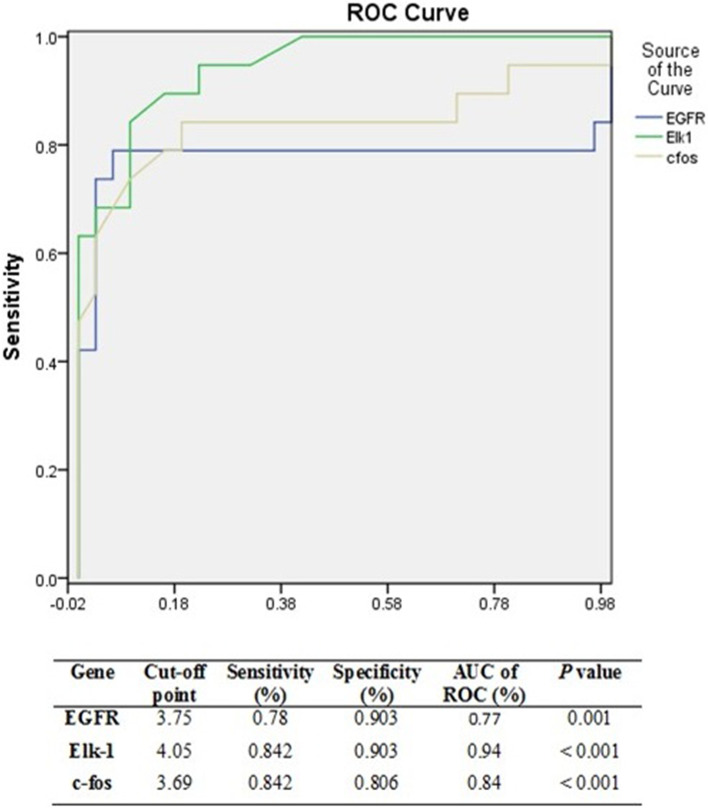
Receiver operating characteristic (ROC) curves were performed to determine the area under the ROC curve (AUC), sensitivity, specificity, and *p*-value to determine the levels of the EGFR, Elk-1, and c-fos that best differentiate the UTUC versus the UBC. The optimal cutoff values were calculated as the marker level that maximizes the sensitivity and specificity

Receiver operating characteristic (ROC) curve was obtained for *EGFR*, *ELK-1*, and *c-fos*. The area under the curve (AUC) for EGFR (0.77), ELK-1 (0.94), and c-fos (0.84) was significantly larger than the reference line (*P* ≤ 0.01). The optimal cutoff values for the investigated biomarkers were determined based on the best balance of sensitivity and specificity. The optimal cutoff values were 3.75, 4.05, and 3.69 for *EGFR*, *ELK-1*, and *c-fos*, respectively (Fig. 1).

### Cell cycle regulator

Comparison between the expression level of mRNA of cell cycle regulator *survivin* and *TP53* showed significantly higher levels of expression in both UTUC and UBC compared to normal tissue (*P* < 0.001). Nevertheless, *RB1* expression showed significantly lower level in both UTUC and UBC compared to normal tissue (*P* < 0.001). UTUC showed significantly higher levels of expression of *survivin* and *TP53* compared to UBC (*P* < 0.001). Meanwhile, *RB1* expression showed comparable results between UTUC and UBC (*P* = 0.133) (Table 3).

**Fig. 2:**
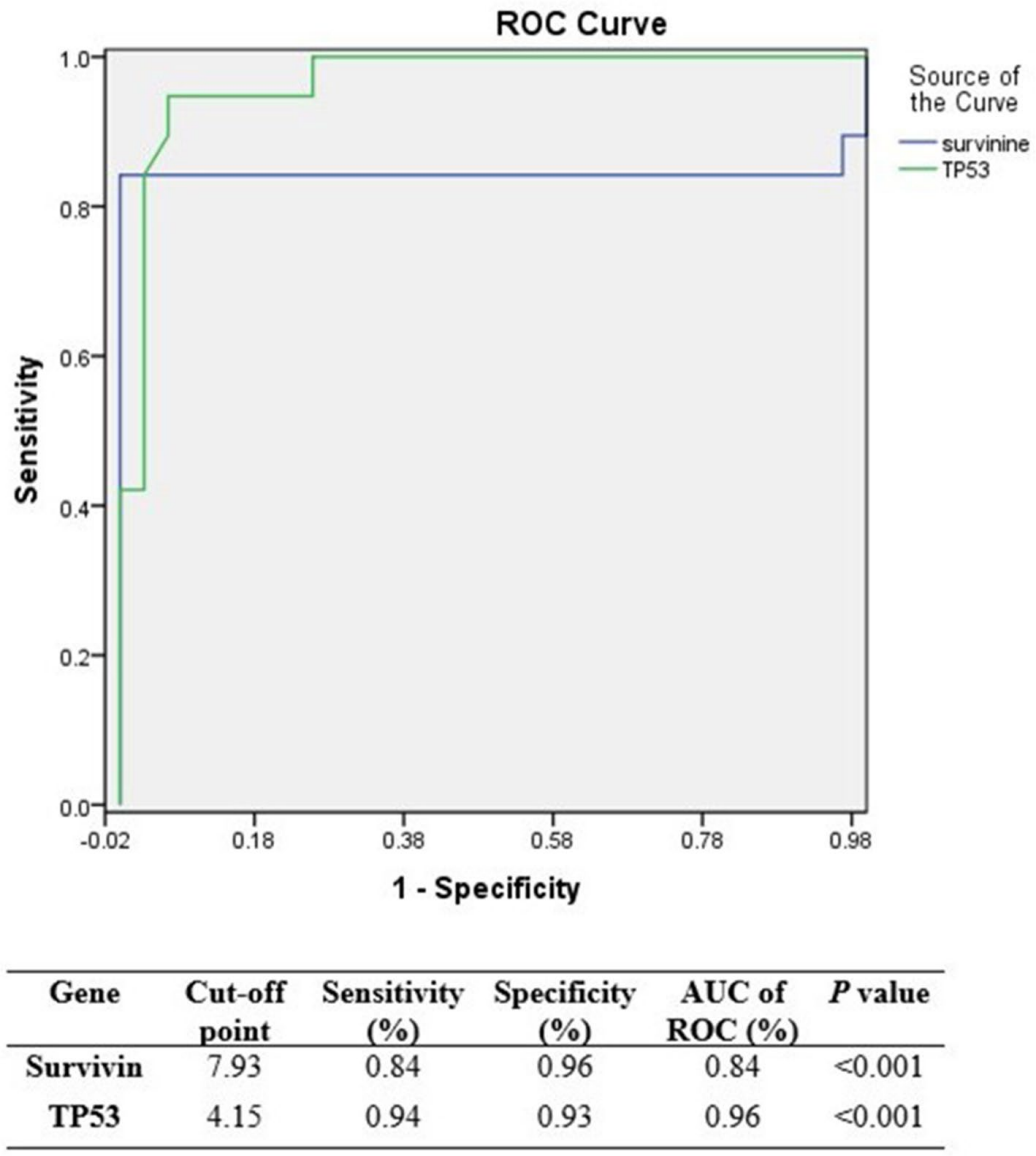
Receiver operating characteristic (ROC) curves were performed to determine the area under the ROC curve (AUC), sensitivity, specificity, and *p*-value to determine the levels of the survivin and TP53 that best differentiate the UTUC versus the UBC. The optimal cutoff values were calculated as the marker level that maximizes the sensitivity and specificity

ROC curve was obtained to *survivin* and *TP53*. The AUC for *survivin* (0.84) and *TP53* (0.96) was significantly larger than the reference line (*P* < 0.01). The optimal cutoff values for the investigated biomarkers were determined based on the best balance of sensitivity and specificity. The optimal cutoff values were 7.93 and 4.15 for *survivin* and *TP53*, respectively (Fig. 2).

### Oncogenic genes

Comparison between the expression level of mRNA of oncogenic genes *FGFR3* and *hTERT* showed significantly higher levels of expression in both UTUC and UBC compared to normal tissue (*P* < 0.001). UTUC showed significantly higher levels of *FGFR3* expression in UTUC compared to UBC (*P* < 0.001). On the other hand, *hTERT* showed comparable results between UTUC and UBC (*P* = 0.395) (Table 3).

**Fig. 3:**
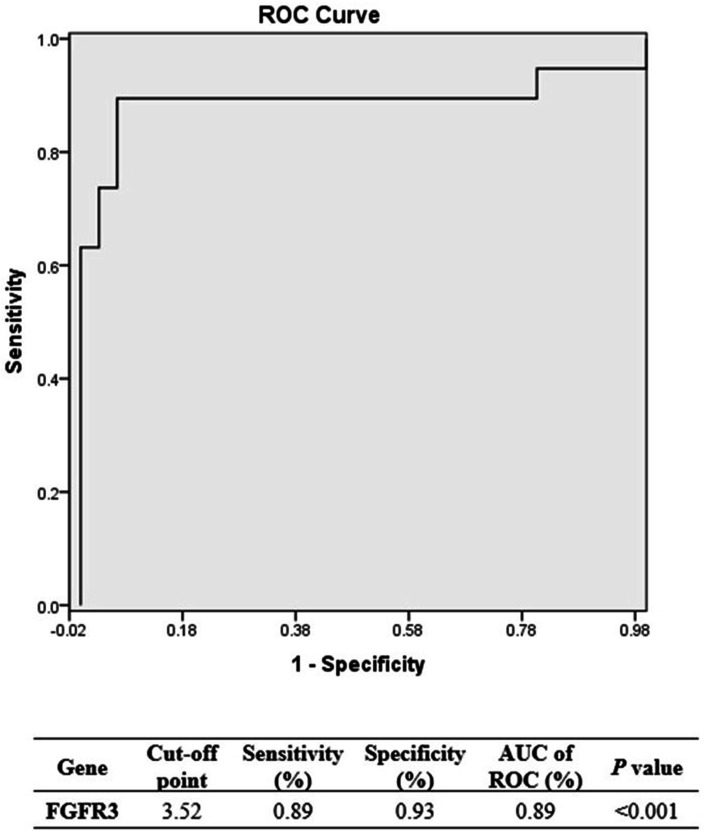
Receiver operating characteristic (ROC) curves were performed to determine the area under the ROC curve (AUC), sensitivity, specificity, and *p*-value to determine the levels of the FGFR3 that best differentiate the UTUC versus the UBC. The optimal cutoff values were calculated as the marker level that maximizes the sensitivity and specificity

ROC curve was obtained for *FGFR3*. The AUC for *FGFR3* (0.89) was significantly larger than the reference line (*P* < 0.01). The optimal cutoff values for the investigated gene were determined based on the best balance of sensitivity and specificity. The optimal cutoff value for *FGFR3* was 3.52 (Fig. 3).

## Discussion

The UTUC and UBC are considered as a disparate twin with distinct putative pathogenetic mechanisms. A molecular study to clarify the discrepancy between them may identify contrasting therapeutic opportunities [4]. The present study investigated (MAPK/ERK) signaling pathway as it plays an important role in the cellular growth differentiation through controlling multiply genes like *EGFR*, *ELK-1*, and *c-fos*.

EGFR has a tyrosine kinase domain which plays an important role in cell signaling pathways involving cell proliferation, apoptosis, angiogenesis, and metastasis through signaling pathways such as MAPK/ERK [15]. Overexpressed or mutant *EGFR* has a critical role in human cancer including the lung, prostate, and urinary bladder [16].

In the present study, *EGFR* was highly expressed in UTUC and UBC tumor specimens compared to normal tissue. This result is consistent with the result reported by Khaled et al. [17] who found that *EGFR* is overexpressed in UBC. However, *EGFR* had significantly higher expression in UTUC than UBC.

ELK1 is a member of transcription factors, regulating the expression of some genes including *c-fos* and sharing a role in cell apoptosis, migration, and invasion. Immunohistochemical study revealed that levels of ELK1, and its activated form, phospho-ELK1, were overexpressed in bladder tumors [18]. In patients with UTUC, phospho-ELK1 were significantly upregulated, compared with corresponding nonneoplastic urothelial tissues [19]. In the present study, *ELK-1* expression was overexpressed in UTUC and UBC tumor tissues compared to normal tissue. On the other hand, *ELK1* expression had significantly higher expression in UTUC than UBC.

c-fos is a proto-oncogene that shares a role in the regulation of cell growth, differentiation, proliferation, transformation, and apoptosis. *c-fos* is significantly expressed in UBC than in adjacent non-cancer tissues [20]. In the present study, *c-fos* expression had significantly higher expression in UTUC and UBC tumor tissues than the adjacent normal. Huhe et al. [21] revealed that c-fos was upregulated in UBC using immunohistochemical (IHC). Interestingly, our study showed that the expression level of *c-fos* was higher in UTUC than UBC.

The present study investigated the cell cycle regulatory genes, as they play a vital role in controlling the replication of apoptosis, and prevents the uncontrolled cell division. The cell cycle is controlled by various genes like *survivin*, *TP53*, and *RB1*.

Survivin is a mitosis regulator and apoptosis inhibitor and promotes angiogenesis. *Survivin* shows significant overexpression in UBC patients and very low to absent levels in non-cancer [22]. Immunohistochemistry shows that survivin protein overexpression is strongly predictive of recurrence and progression of BC [23]. Altered *survivin* expression was observed in 39.3% of UTUC tumors and was significantly associated with worse clinicopathological features as advanced stages, lymph node metastases, and lymphovascular invasion [10]. In our trial, *survivin* was overexpressed in both UTUC and UBC tumor tissues compared to the adjacent normal tissue. It was observed that the level of the *survivin* expression was significantly higher in UTUC than UBC.

TP53 maintains the genomic stability and activation of cell cycle exit/apoptosis through working as a tumor suppressor. The prevalence of *TP53* alterations increases with UBC stage and grade [24]. *TP53* was more frequently altered in UBC; however, it was only present in high-grade and high-stage UTUC [25]. *TP53* was the second most frequently altered genes in both in UBC and UTUC (58% vs 49%, *p* = 0.04), respectively [4]. In our study, *TP53* is overexpressed in both types of tumor compared to the normal tissue. This result agrees with Khaled et al. [17] who reported that *TP53* was overexpressed in UBC. Also in our study, it was observed that the *TP53* was significantly higher in UTUC than UBC.

The RB1 is a key molecular component that regulates cell cycle progression. It controls the G1-S transition in the cell cycle by integrating growth-promoting and suppressing signals. Cancer cells often have mutations in this pathway, which lead to uncontrolled tumor development [26]. In our study, *RB1* mRNA expression was low in both UTUC and UBC compared to normal tissue. This result agrees with Khaled et al. [17] who reported that *RB1* is negatively expressed in UBC. We also showed no significant difference between expression of *RB1* in UTUC and UBC.

The present study investigated the oncogenic genes that play a role in cell growth and proliferation or inhibition of apoptosis that are controlled by multiple genes like *FGFR3* and *hTERT*.

FGFR3 is a transmembrane tyrosine kinase receptor with auto-phosphorylation activity that controls a variety of physiological processes such as proliferation, differentiation, migration, and apoptosis, and its structural activation is linked to several diseases, including cancer [27].

Kang et al. [28] reported that *FGFR3* is overexpressed in UBC tissue. In the present study, *FGRF3* was found to be overexpressed in UTUC and UBC compared to the adjacent normal tissue. *FGFR3* had significantly higher expression in UTUC than UBC.

Telomerase is a ribonucleoprotein enzyme that keeps the telomere length constant by adding telomeric repeats (TTAGGG) to the end of the telomere. The gene *hTERT* codes for telomerase. In normal human cells, telomerase activity is inhibited, and telomeres are increasingly reduced after each replication cycle, resulting in cell senescence and death [29]. In over 85–90% of malignant cancer cells, telomerase is reactivated and overexpressed, enabling them to survive and proliferate uncontrollably [30]. *hTERT* was expressed in high level in UTUC and UBC tumor tissue compared to the adjacent normal. Such result was observed in the work of Wu et al. [31] who reported that *hTERT* was highly expressed in UTUC tissue. In our study, the comparison between expression of *hTERT* UTUC and UBC had no significant difference.

The significant expression of *EGFR*, *ELK1*, *c-fos*, *survivin*, *TP53*, and *FGFR3* in UTUC could explain its aggressive and invasive attitude than UBC [5]. The results of this study showed that *EGFR*, *ELK1*, *c-fos*, *survivin*, *TP53*, and *FGFR3* are very likely to be involved in the occurrence and development of UTUC. Our study provided references for the molecular etiology of the aggressiveness and invasiveness of UTUC.

The study is limited by having a relatively small sample size and investigating only three molecular pathways of carcinogenesis. Researchers are invited to reproduce this experiment with large sample size and more in-depth studies of large number of molecular pathways. Such larger prospective studies are warranted to consolidate data of our initial results of this preliminary report. Moreover, we advise future researcher to investigate the correlation between the different genes and clinical status of the patient including tumor stage, grade, lymph node status, and metastasis. It might be advisable to use the abnormal expression of the studied genes in gene or target therapy in future studies.

## Conclusions

Results of the present study showed clear evidence that there is a molecular difference between UTUC and UBC despite they share the same origin. This molecular difference could be the reason that UTUC is more aggressive than UBC.

## Data Availability

All data and material are available if requested.

## Abbreviations

AUC: Area under the curve
BC: Bladder cancer
c-DNA: Complementary DNA
EGFR: Epidermal growth factor receptor
FGFR3: Fibroblast growth factor receptor
IHC: Immunohistochemical
RB1: Retinoblastoma
ROC: Receiver operating characteristic
RT-PCR: Real-time polymerase chain reaction
TCGA: The Cancer Genome Atlas
UBC: Urothelial bladder cancer
UC: Urothelial carcinoma
UTUC: Upper tract urothelial carcinoma
